# Expression and significance of lysyl oxidase-like 1 and fibulin-5 in the cardinal ligament tissue of patients with pelvic floor dysfunction

**DOI:** 10.7555/JBR.27.20110142

**Published:** 2012-09-06

**Authors:** Yang Zhou, Ouyang Ling, Li Bo

**Affiliations:** Department of Obstetrics and Gynecology, Shengjing Hospital of China Medical University, Shenyang, Lioaning 110000, China.

**Keywords:** pelvic organ prolapse, stress urinary incontinence, pelvic floor dysfunction, lysyl oxidase-like 1, fibulin-5

## Abstract

Pelvic organ prolapse (POP) is a disabling disorder in women characterized by a loss of pelvic floor support, leading to the herniation of the uterus into or through the vagina. POP is a complex problem that likely involves multiple mechanisms with limited therapies available, and is associated with defects in connective tissue including elastic fibers. This study was designed to investigate the expression of fibulin-5 and lysyl oxidase-like 1 (LOXL1) in the cardinal ligament in samples taken from the POP group compared to the non-POP group. Specimens were obtained during abdominal hysterectomy from the cardinal ligament of 53 women with POP and 25 age- and parity- matched women with non-POP among post-menopausal women with benign gynecologic pathology. Protein expression was evaluated using the immunohistochemical staining method. For statistical analyses, chi-square test and Spearman's correlation were used with the statistical package SPSS13.0 system. Our results showed that both fibulin-5 and LOXL1 expressions were decreased in the cardinal ligament in the POP group compared to the non-POP group (*P* < 0.05). The expression of fibulin-5 and LOXL1 were correlated closely with the stage of POP, accompanied by stress urinary incontinence and frequency of vaginal delivery (*P* < 0.05), but had no relationship with post-menopausal state (*P* > 0.05). The expression of fibulin-5 was positively associated with LOXL1 in POP (*P* < 0.05). We conclude that changes in fibulin-5 and LOXL1 expression may play a role in the development of POP.

## INTRODUCTION

Pelvic floor dysfunction (PFD), a disease with anatomical and/or functional abnormalities of pelvic organs due to weakened supporting tissues of the pelvic floor and dislocation of pelvic organs, is common in women and can considerably interfere with their quality of life. This disease includes pelvic organ prolapse (POP) and stress urinary incontinence(SUI). PFD may significantly impair women's quality of life[1]. Approximately 11% of all women will undergo surgery for urinary incontinence or POP by age 80 years, but the actual prevalence of these disorders is estimated to be as high as 50% of all parous women[2]. But till now, the pathophysiology of pelvic floor disorders remains not well understood. Increasing age and vaginal multiparity are the main commonly accepted factors. The hypothesis of a defect of the connective tissues of the pelvic floor with aging due to collagen deficiency and/or elastic fiber degradation is often highlighted. Recent studies suggest that elastic fibers and collagens are major ingredients of the pelvic connective tissues[3]. As reported by Jung *et al*.[4], fibulin-5 and lysyl oxidase-like 1 (LOXL1) play an essential role in the synthesis and assembly of elastic fibers in the uterosacral ligament from women with advanced POP compared with controls. Tremollieres[3] demonstrated signs of elastinopathy, including the development of POP in the postpartum mice with null mutation in the gene encoding LOXL1 or fibulin-5. LOXL1 is a copper-dependent amine oxidase that plays a critical role in the biogenesis of connective tissue matrix by crosslinking the extracellular matrix (ECM) proteins, collagen and lysyl oxidase. These cross-links are essential for the tensile strength of collagens that are necessary for the structural integrity and function of connective tissues. LOXL1 is the key enzyme for the maturation of amnion, using copper as an essential active co-factor[5],[6]. Fibulin-5 is crucial for lysyl oxidase assembly and is believed to act as a bridge between cells and tropolysyl oxidase for effective cross-linking and assembly of tropolysyl oxidase into mature elastic fibers[7]. The aim of this study was to explore the relationship of fibulin-5 and LOXL1 expression in the cardinal ligament tissue of women with pelvic floor dysfunction in the pathogenesis of POP.

## SUBJECTS AND METHODS

### Subjects

Specimens were obtained prospectively during abdominal hysterectomy from 53 women with advanced POP and 25 non-POP controls matched to the study group. All the women underwent operations at Shengjing Hospital of China Medical University between November 2008 and June 2010. Informed consent was obtained from enrolled patients before sample collection. The protocol was approved by the Ethics Committee of Shengjing Hospital. Women with POP (stage III–IV) underwent abdominal hysterectomy for their advanced disease. The controls matched for age and parity were selected from post-menopausal women with benign gynecologic pathology and were subject to abdominal hysterectomy due to endometrial pathology, benign ovarian tumor or uterine myomas. Patients with previous pelvic surgery, connective tissue disease, or history of cancer were excluded.

All patients were assessed with a standard questionnaire and physical examination before surgery. This questionnaire included questions regarding age, parity, body mass index, post-menopausal status, prior history of hormone replacement therapy, previous gynecologic histories of genetic diseases, gynecologic operation or anti-incontinence operation, and medical histories of connective tissue disease, hypertension, diabetes mellitus, chronic obstructive pulmonary disease and lumbar disc herniation. POP was quantified according to the International Continence Society's Pelvic Organ Prolapse Quantification (POP-Q) system[8]. Pelvic examinations were performed by the same examiner, and 19 patients were categorized in grade II and 34 patients in grade III and IV by severity. Patients aged 51 to 63 years were enrolled with menstrual status as pre-post-menopausal (*n* = 5) and post-menopausal (*n* = 48). Thirty-five patients were complicated with stress urinary incontinence (SUI). Diagnostic criteria of SUI are presented as follows: 1) Patients who reported urinary overflow induced by increased abdominal pressure; 2) Bladder detrusor instability was excluded by urodynamic examination; 3) Abdominal Leak Point Pressure (ALPP)> 60 cm H_2_O; 4) Presence of normal neurological function of the pelvic floor and urine analysis. Patients in the control group (*n* = 25) received hysterectomy for gynecological benign tumor in the same period. These patients did not present with clinical symptoms of urinary incontinence (SUI and POP were excluded by pelvic examination), and were not administered with drugs containing hormones within three months prior to enrollment (Female hormone related diseases such as uterine endometriosis and functional ovarian tumors were excluded pathologically). No rheumatoid arthritis, hyperthyroidism and hyperparathyroidism or other diseases that affected collagen metabolism were found in the case group and control group. There were no statistically significant differences in age, number of vaginal deliveries, body mass index and menopause between the POP and control groups (*P* > 0.05) (shown in ***Table 1***).

Tissue samples were obtained during hysterectomy in the operation room. An approximately 2.0 cm×1.0 cm sized sample from the cardinal ligament was harvested immediately before hysterectomy from the enthesis of the ligament into the cervix where it has been indicated as an exclusively important portion in providing support for the uterus. The specimens collected were snap frozen in liquid nitrogen and stored at –80°C.

**Table 1:** Comparison of general conditions between women with or without pelvic organ prolapse (POP)

Group	Number of cases	Age(year, x±s)	Mean number of vaginal deliveries	BMI(kg/m^2^, x±s)	Menopause
					Numer of cases (%)
POP	53	57±6	3	24±3	48(91)
Control		55±3	2	24±4	23(92)
*P*	25	> 0.05	> 0.05	> 0.05	> 0.05

BMI: body mass index; POP: pelvic organ prolapse.

### Immunohistochemistry staining

Four-micron thick sections were prepared from the paraffin-embedded tissues. Immunohistochemical staining was performed by the streptavidin-peroxidase (S-P) method (Ultrasensitive™ MaiXin, Fuzhou, China). The primary antibodies used in this study were anti-human fibulin-5 and anti-human LOXL1 mouse monoclonal antibody (both diluted 1:100, Santa Cruz Biotechnology, Santa Cruz, CA, USA), S-P kit were purchased from Zhongshan Biotechnology Corporation. DAB agent kit and horseradish peroxidase labeled goat-anti-mouse IgG were bought from Beijing Zhongshan Golden Bridge Biotechnology Corporation, Beijing, China. For the negative control, all the procedures were the same as those of the test section with the exception of the primary antibodies that were replaced by PBS.

**Fig. 1:**
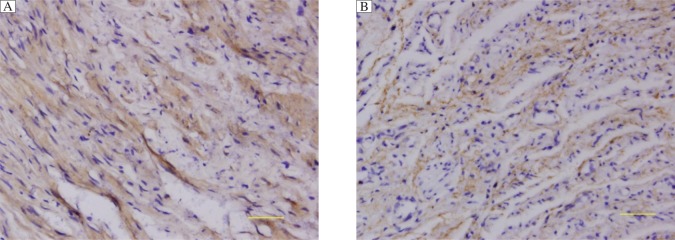
Expressions of lysyl oxidase-like 1 (A) and fibulin-5 (B) by immunohistochemistry (magnification: ×400; scale bar: 50 µm). Positive expression was indicated by brown-yellow granules in the cardinal ligament tissues of patients with pelvic organ prolapse.

The expression of fibulin-5 and LOXL1 detected by staining was graded by the percentage of immunoreactive ECM. All immunostained sections were scored independently by two authors who were blinded to the study information of the sections. Cases with discrepancies were jointly re-evaluated by the two investigators until a consensus was obtained. The sections were evaluated at 10 random areas at 400 magnification.(shown in ***Fig. 1***)

### Statistical analysis

All the statistical analyses were carried out using statistical package SPSS13.0 (SPSS Inc., Chicago, IL, USA). The chi-square test was used to determine the correlation between fibulin-5 or LOXL1 expression and clinicopathological factors. The relationship between fibulin-5 and LOXL1 was analyzed by Spearman's rank correlation coefficient. Values of *P* < 0.05 were considered statistically significant.

## RESULTS

### Relationship between the expression of fibulin-5 and pathological features in the cardinal ligaments.

Fibulin-5 was positively expressed in the cardinal ligament tissues of 23% (12/53) of patients for the POP group versus 56% (14/25) in the control group with significantly decreased fibulin-5 levels in the POP group (*P* = 0.005, ***Fig. 1***). Furthermore, in the POP group, the expression of fibulin-5 in women with stage III/IV POP [12% (4/34)] was downregulated more significantly than those with stage II POP [42%(8/19)] (*P* = 0.020). However, no statistical difference was found between post-menopausal patients [38% (18/48)] and non- menopausal patients (1/5) (*P* > 0.05) and between patients with vaginal deliveries≤2 [18%(4/22)] and >2 [23% (7/31)] (*P* > 0.05). The positive rate was also less significantly decreased in the POP group with SUI [26%(9/35)] than the non-SUI POP [33%(6/18)] group (*P* > 0.05) (***Table 1*** and ***Table 2***).

### Expression of LOXL1 and relationship with pathological features.

The LOXL1 positive expression rate in the cardinal ligament of pre-menopausal and post-menopausal POP group was 2/5 and 12/48, respectively (*P* > 0.05). Significant difference was found in the expression of LOXL1 in the cardinal ligament between the POP group [26%(14/53)] and the control group [56% (14/25)] (χ^2^ = 7.126, *P* < 0.05). Patients with lower LOXL1 expression exhibited a more advanced stage of POP (II *vs* III+IV, *P* = 0.027). There was no significant difference in LOXL1 expression in the cardinal ligament between SUI [31%(11/35)] and non-SUI subgroups with POP [22%(4/18)] (χ^2^ = 0.496, *P* > 0.05). Similar results were found on the vaginal delivery frequency of the POP group (χ^2^ = 0.576, *P* > 0.05, ***Table 2***).

**Table 2:** Expression of fibulin-5 and LOXL1 in the cardinal ligament between women with or without pelvic organ prolapse (POP)

Group	Total cases	LOXL1 positivity				Fibulin-5 positivity			
		Cases	Percentage(%)	χ^2^	*P*	Cases	Percentage(%)	χ^2^	*P*
POP group	53	14	26			12	23		
POP-Q				5.537	0.027			5.972	0.020
Stage II	19	9	47			8	42		
Stage III+IV	34	5	15			4	12		
Menopause				0.524	0.599			0.603	0.643
Yes	48	12	25			18	38		
No	5	2	2/5*			1	1/5*		
No. of vaginal deliveries				0.576	0.544			0.151	0.746
≤2	22	5	23			4	18		
> 2	31	10	32			7	23		
SUI									
No	18	4	22	0.496	0.539	6	33	0.340	0.748
Yes	35	11	31			9	26		
Control group	25	14	56	7.126	0.011	14	56	8.506	0.005

“*” means total cases are less than 10, when we described with fraction instead of percentage. SUI: stress urinary incontinence.

### Relationship between fibulin-5 and LOXL1 expression in the POP group.

Co-expression of fibulin-5 and LOXL1 in the cardinal ligament tissues was found in 9 patients with POP while negative expression of both fibulin-5 and LOXL1 was demonstrated in 24 patients. Relationship between LOXL1 and fibulin-5 was analyzed by Spearman test in the POP group (*P* < 0.05), indicating a positive relationship between LOXL1 and fibulin-5 (***Table 3***).

**Table 3:** Relationship between fibulin-5 and LOXL1 expression in the cardinal ligament of patients with pelvic organ prolapse

LOXL1	Fibulin-5		*P*
	Positive	Negative	
Positive	9	14	0.017
Negative	6	24	

BMI: body mass index.

## DISCUSSION

POP is a multifactorial disease in old women. Several environmental risk factors that could cause qualitative and quantitative changes in the connective tissue that provides support for the lower pelvic organs and result in POP[9],[10]. The connective tissues are composed of ECMs, including collagen, proteoglycans, LOXL1 and glucoprotein. Changes in the content and/or structure of these ingredients may induce decreased elasticity and tensile strength and weakened pelvic floor support, finally resulting in POP[11]. Several studies have reported that elastic fibers are decreased in the cardinal ligaments or the vaginal wall with increased elastolytic activity in POP patients[12],[13],[14].

The synthesis and assembly of elastic fibers is a complicated process that is not fully understood. Tropoelastins are secreted primarily from fibroblasts and smooth muscle cells. Microfibrils consist of several proteins that form a scaffold on which elastin deposits before it is displaced to the periphery of the growing fiber. This scaffold is cross-linked by one or more lysyl oxidases[15]. Fibulin-5[16] is also known as FBLN-5, DANCE, or EVEC, which is a member of the fibulin family in ECM[16]. It is extensively distributed in LOXL1-rich tissues. Human *fibulin-5* gene is located in 14q32.1, and the carboxyl portions of fibulin-5 are 6 epidermal growth factor-like regions that can bind with calcium. One of these regions contain arginine-glycine-aspartic acid motif that can combine with integrin, functioning to mediate endothelial cell adhesion and anchor the original LOXL1 on the cell surface by binding to integrin αγβ3, αγβ5 and α9β1 on cell surface, which is crucial for the formation of elastic fibers[17]. Drewes et al.[18] found that 3-month-old *fibulin-5* gene knockout mice did not develop vaginal wall prolapse and larger reproductive hiatus. Our study showed decreased fibulin-5 expression in POP connective tissue and a significantly lower fibulin-5 protein expression in the stage III+IV group than stage II (*P* < 0.05), indicating that fibulin-5 may have an important role in the occurrence and development of POP.

LOXL1[19] is an extracellular copper-dependent monoamine oxidase, which can catalyze the crosslinking of lysine residues in collagen and elastic fibers in the ECM, promoting the maturation of collagen and elastin to maintain normal structure and function of ECM. It is a key enzyme in the crosslinking between collagen and elastic fibers and also plays an important role in the initial stage of the process where collagen and LOXL1 soluble monomers change into insoluble fibers[20]. Liu et al.[21] found that *LOXL1* knockout mice can develop POP. Kobak et al.[22] reported that mRNA expression of *LOXL1* was significantly reduced in the sacral ligament in patients with POP, creating fragile immature collagen and elastic fibers, consequently leading to a weakened support of the pelvic floor. In agreement with the results of Kobak, our study also showed that LOXL1 expression in the cardinal ligament of the POP group was significantly lower than that of the control group (*P* < 0.05). This suggests that LOXL1 may play a role in the pathogenesis of POP. In addition, we found no significant differences between the pre-menopausal and post-menopausal groups in our study.

POP and SUI usually are present together. It is reported that in clinical settings, about 60% POP is complicated with SUI, and 80% or more of SUI patients may be combined with different degrees of POP. It is generally believed that both conditions have similar etiology and pathogenesis[18]. Moreover, our experiment found that LOXL1 and fibulin-5 expression in the cardinal ligament of POP patients with SUI was less significantly reduced. Furthermore, we conducted a correlation analysis between LOXL1 and fibulin-5 expression in the cardinal ligament by Spearman analysis. The result indicates that these two genes have the same trend at the protein level, both showing a significant positive correlation (*P* < 0.05), indicating that decreased expression of fibulin-5 and LOXL1 may cooperatively lead to synthetic dysfunction of elastin and/or unstable elastic fiber. As a result, the elasticity of ECM in pelvic floor supporting tissue decreases, resulting in the occurrence of POP eventually.

In summary, POP is a multifactorial disease, and our study has revealed that LOXL1 and fibulin-5 protein expression is downregulated in the pelvic connective tissue of POP patients, and is related to POP grade, indicating that in pelvic tissues, decreased LOXL1 and fibulin-5 may be very important for weakened pelvic floor support and urine control capacity, which may eventually lead to the occurrence and development of POP.

This study examined fibulin-5 and LOXL1; however, the specific mechanism of LOXL1 and fibulin-5 participating in the occurrence and development of POP is unclear. Future studies are needed to delineate the underlying mechanisms.
